# Long-lasting masculinizing effects of postnatal androgens on myelin governed by the brain androgen receptor

**DOI:** 10.1371/journal.pgen.1007049

**Published:** 2017-11-06

**Authors:** Charly Abi Ghanem, Cindy Degerny, Rashad Hussain, Philippe Liere, Antoine Pianos, Sophie Tourpin, René Habert, Wendy B. Macklin, Michael Schumacher, Abdel M. Ghoumari

**Affiliations:** 1 U1195 Inserm and Universities Paris-Sud and Paris-Saclay, 80 rue du Général Leclerc, Kremlin-Bicêtre, France; 2 Department of Neurosurgery, Institute for Translational Neuromedicine, University of Rochester, Rochester, NY, United States of America; 3 U566 Inserm, CEA, Universities Paris-Diderot and Paris-Sud, Fontenay aux Roses, France; 4 Department of Cell and Developmental Biology, University of Colorado, Aurora, CO, United States of America; The Rockefeller University, UNITED STATES

## Abstract

The oligodendrocyte density is greater and myelin sheaths are thicker in the adult male mouse brain when compared with females. Here, we show that these sex differences emerge during the first 10 postnatal days, precisely at a stage when a late wave of oligodendrocyte progenitor cells arises and starts differentiating. Androgen levels, analyzed by gas chromatography/tandem-mass spectrometry, were higher in males than in females during this period. Treating male pups with flutamide, an androgen receptor (AR) antagonist, or female pups with 5α-dihydrotestosterone (5α-DHT), revealed the importance of postnatal androgens in masculinizing myelin and their persistent effect into adulthood. A key role of the brain AR in establishing the sexual phenotype of myelin was demonstrated by its conditional deletion. Our results uncover a new persistent effect of postnatal AR signaling, with implications for neurodevelopmental disorders and sex differences in multiple sclerosis.

## Author summary

Sex differences in brain structure are of great scientific and medical interest because the incidence and progress of many neurological and psychiatric disorders differ between males and females. They affect neural networks and also the myelin sheaths that insulate and protect axons and thus allow the rapid conduction of electrical impulses. In the central nervous system, myelin is formed by a particular type of cells named oligodendrocytes. In the male mouse brain, the density of oligodendrocytes is greater and myelin sheaths are thicker when compared with females. We show that these sex differences in myelin result from the long-lasting actions of androgens in males during their first 10 postnatal days. Importantly, the postnatal masculinizing effects of androgens involve brain androgen receptors as shown by the use of pharmacological and genetic tools. These findings are important for understanding sex-related differences in the susceptibility and progression of demyelinating diseases such as multiple sclerosis. They also reveal a so far unknown role of androgen receptor signaling in sexual differentiation of the brain.

## Introduction

The incidence and clinical course of many neurological disorders differ between sexes, and elucidating the underlying biological basis has become a high priority challenge [1, 2]. The potential impact of sex differences in brain structure has long been neglected. They were indeed believed to be restricted to specific brain regions, in particular those involved in reproductive functions [3]. This concept has changed with recent neuroimaging studies uncovering sex differences in neuronal connectivity across the entire brain [4, 5]. Moreover, structural sex differences in the human brain are shaped by fetal testosterone [6].

Rodent models have provided valuable insights into mechanisms leading to sex differences in brain structure and function. They can be reversible and only caused by the temporary actions of sex-specific hormones [7]. Alternatively, sex dimorphism in brain may be persistent and result from developmental processes, including the masculinizing actions of testicular testosterone during sensitive perinatal and postnatal periods, and the shaping of neuronal circuits by sex chromosome-linked genes, epigenetic factors and the hormonal environment [8–11]. In mice and rats, neural circuits are sensitive to the persistent differentiating (organizational) effects of gonadal steroids around birth and during a postnatal period which may extent to 4 weeks [3, 12]. During the perinatal period, the male brain is exposed to the masculinizing effects of a transient surge of testicular testosterone, driven by kisspeptin and gonadotropin released by hypothalamic neurons [13]. In both rats and mice, the aromatization of testosterone to estradiol plays an important role in masculinization of the brain [14–16]. However, disrupting androgen receptor (AR) signaling also interferes with the process of hormone-dependent sexual differentiation of the brain [17, 18]. The respective roles of estrogen receptor (ER) and AR signaling are not completely understood. In mice, AR are sparse in the brain at the time of the neonatal testosterone surge, and their expression only increases by postnatal day 4 (P4) [17, 19]. For this reason, it can be presumed that estrogens play a major role in the organizational effects of neonatal testosterone, when brain ER and aromatase are highly expressed, and that the role of AR signaling may become more important during postnatal brain development [17]. However, aromatase knockout male mice, developmentally deprived of their brain estrogens, show normal coital behavior following adult hormone treatment [20]. It is likely that respective organizational functions of androgens and estrogens are dependent on brain functions and differ between species.

Intriguingly, a sexual dimorphism affecting the density of oligodendrocytes, the myelin forming glial cells of the central nervous system (CNS), and the structure of myelin has been reported in adult mice and rats [21]. The density of oligodendrocytes was found to be 20–40% greater in adult males compared with females in the corpus callosum and other white matter tracts of the CNS. Moreover, the expression of myelin basic protein (MBP) and proteolipid protein (PLP), two myelin-specific proteins, was significantly greater in males. Interestingly, the sexual dimorphism of oligodendrocytes and myelin was sensitive to long-term castration, over 3 months. Indeed, the density of oligodendrocytes was decreased and became comparable to the one observed in females, pointing to a possible role of testicular secretions [21]. However, a persistent organizational effect of neonatal androgens on myelin appeared highly unlikely, as oligodendrocyte progenitors arise in the rodent cerebral cortex and corpus callosum and begin to differentiate into myelinating oligodendrocytes between the first and second postnatal week [22].

Herein, we report the unexpected observation that myelin is in fact sexually differentiated in mice by postnatal androgens and AR signaling. We establish that sex differences in myelin are already present at postnatal day 10 (P10) using a transgenic mouse line selectively expressing the enhanced green fluorescent protein (EGFP) in the oligodendroglial cell lineage [23]. Furthermore, using gas chromatography coupled to tandem mass spectrometry (GC-MS/MS) [24, 25], we report that brain levels of testosterone and 5α-DHT, both endogenous agonist ligands of the AR, are significantly higher in males when compared with females between postnatal days P0 and P10, We also show persistent effects of postnatal androgens on the density of oligodendrocytes and the structure of the myelin sheaths by postnatal pharmacological treatments. Finally, we demonstrate a key role of brain AR in the structural phenotype of myelin by specifically deleting the receptor in neural cells of the CNS. The role of AR in determining the structure of myelin was further strengthened in genetic male mice with the testicular feminization mutation (Tfm), which lack functional AR. These findings provide new insights into the sexual differentiation of the brain, moving persistent sex differences from neurons to myelin and uncovering new long-lasting effects of postnatal AR signaling.

## Results

### Sex differences in the density of corpus callosum oligodendrocytes already appear between postnatal days 5 and 10

**Fig 1:**
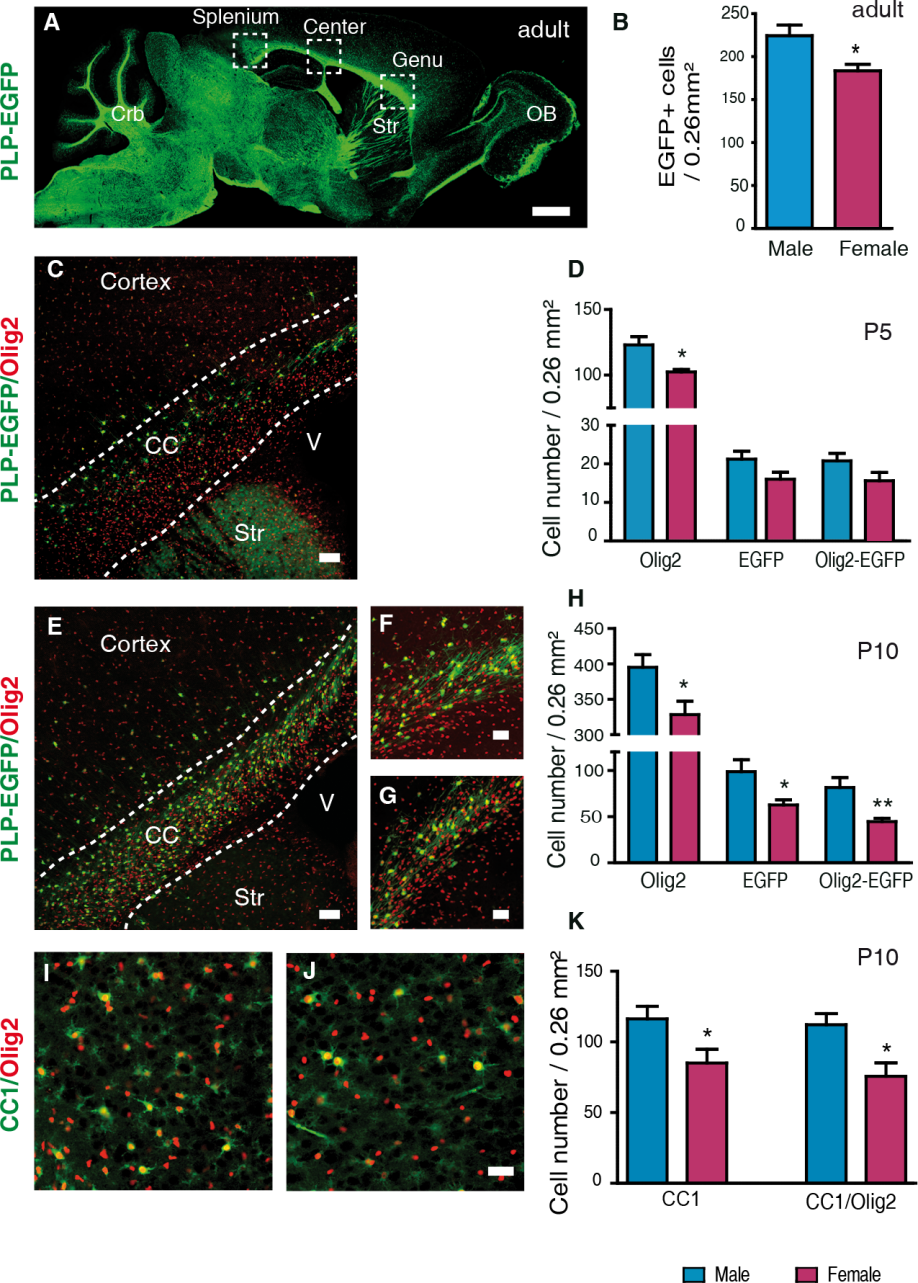
The density of oligodendroglial cells becomes sexually differentiated between P5 and P10. (A) Sagittal section showing EGFP expression in the adult PLP-EGFP mouse brain. White matter tracts display intense green fluorescence, including the three subregions of the corpus callosum: genu, center and splenium. Crb = cerebellum; OB = olfactory bulb; Str = striatum.(B) Comparison of the densities of EGFP^+^ oligodendroglial cells in corpus callosum (CC) between adult male and female PLP-EGFP mice (n = 5). (C) Immunostaining of Olig2^+^ gliogenic progenitors (red) and EGFP^+^ oligodendroglial cells (green) on a sagittal brain section of a PLP-EGFP male mouse at P5. CC = corpus callosum; Str = striatum; V = third ventricle. (D) Comparison of the densities of Olig2^+^, EGFP^+^ and Olig2^+^/EGFP^+^ double-positive cells in corpus callosum between P5 male and female PLP-EGFP mice (n = 5). Immunostaining of Olig2^+^ cells (red) and EGFP^+^ cells (green) on sagittal brain sections at P10 of (E and F) a male and (G) a female. CC = corpus callosum; Str = striatum; V = third ventricle. (H) Comparison of the densities of Olig2^+^, EGFP^+^ and Olig2^+^/EGFP^+^ double-positive cells in corpus callosum between P10 males and females PLP-EGFP mice (n = 9). (I and J) Immunostaining of Olig2^+^ cells (red) and CC1^+^ mature oligodendrocytes (green) on sagittal corpus callosum sections at P10 of (I) a male and (J) a female C57Bl/6 mouse. (K) Comparison of the densities of CC1^+^ oligodendrocytes and CC1^+^/Olig2^+^ double-positive cells in corpus callosum at P10 between males and females (n = 5). Cell densities are presented as means ± SEM. Significance was calculated using two-tailed Student’s t test (**p < 0.01; *p < 0.05 when compared to the corresponding male group). Scale bars: (A) 1 mm; (C and E) 50 μm; (F-J) 20 μm.

To study the developmental origin and hormonal determinants of the sexual dimorphism of oligodendrocytes and myelin, we used a transgenic mouse line expressing the enhanced green fluorescent protein (EGFP) driven by the *PLP* promoter (PLP-EGFP mouse). In this mouse, EGFP is selectively expressed in cells of the oligodendroglial lineage throughout the brain (Fig 1A) [23]. As expected, the density of fluorescent oligodendroglial cells was about 20% higher in the corpus callosum of adult male mice when compared with females (Fig 1B).

As the sexual dimorphism of oligodendrocytes and myelin has been previously demonstrated only in adult rodents [21], we investigated whether it originates early during postnatal brain development. We observed a 17% higher density of cells expressing the transcription factor Olig2 (oligodendroglial lineage marker) in PLP-EGFP male mice when compared with females as early as postnatal day 5 (P5, Fig 1C and 1D). However, the density of EGFP^+^ and EGFP^+^/Olig2^+^ oligodendroglial cells did not significantly differ between sexes. At this early stage, Olig2 is still expressed in progenitors of both astrocytes and oligodendrocytes [26, 27]. Only 20% of the Olig2^+^ glial progenitors were also EGFP^+^, which would be expressed in the maturing cells of thus belonging to the oligodendroglial lineage. On the other hand, all EGFP^+^ cells expressed Olig2, confirming the restricted expression of the fluorescent marker [28].

Between P5 and P10, there was an increase in the density of Olig2^+^, EGFP^+^ and EGFP^+^/Olig2^+^ cells in the corpus callosum of both sexes. At P10, the density of all these markers was 20% higher in males when compared with females (Fig 1E–1H). Likewise, the number of cells labelled with the CC1 antibody directed against adenomatous polyposis coli, an accepted marker of differentiated oligodendrocytes [29], and of Olig2^+^/CC1^+^ co-expressing oligodendrocytes was higher in males than in females at P10 (Fig 1I–1K). Thus, the density of oligodendroglial cells becomes sexually dimorphic between P5 and P10 in the mouse corpus callosum. Moreover, the number of EGFP+ cells in the transgenic mice closely approximates the number of CC1+ cells in wild type mice (Fig 1H–1K).

### Corpus callosum myelin differs between males and females at postnatal day 10

**Fig 2:**
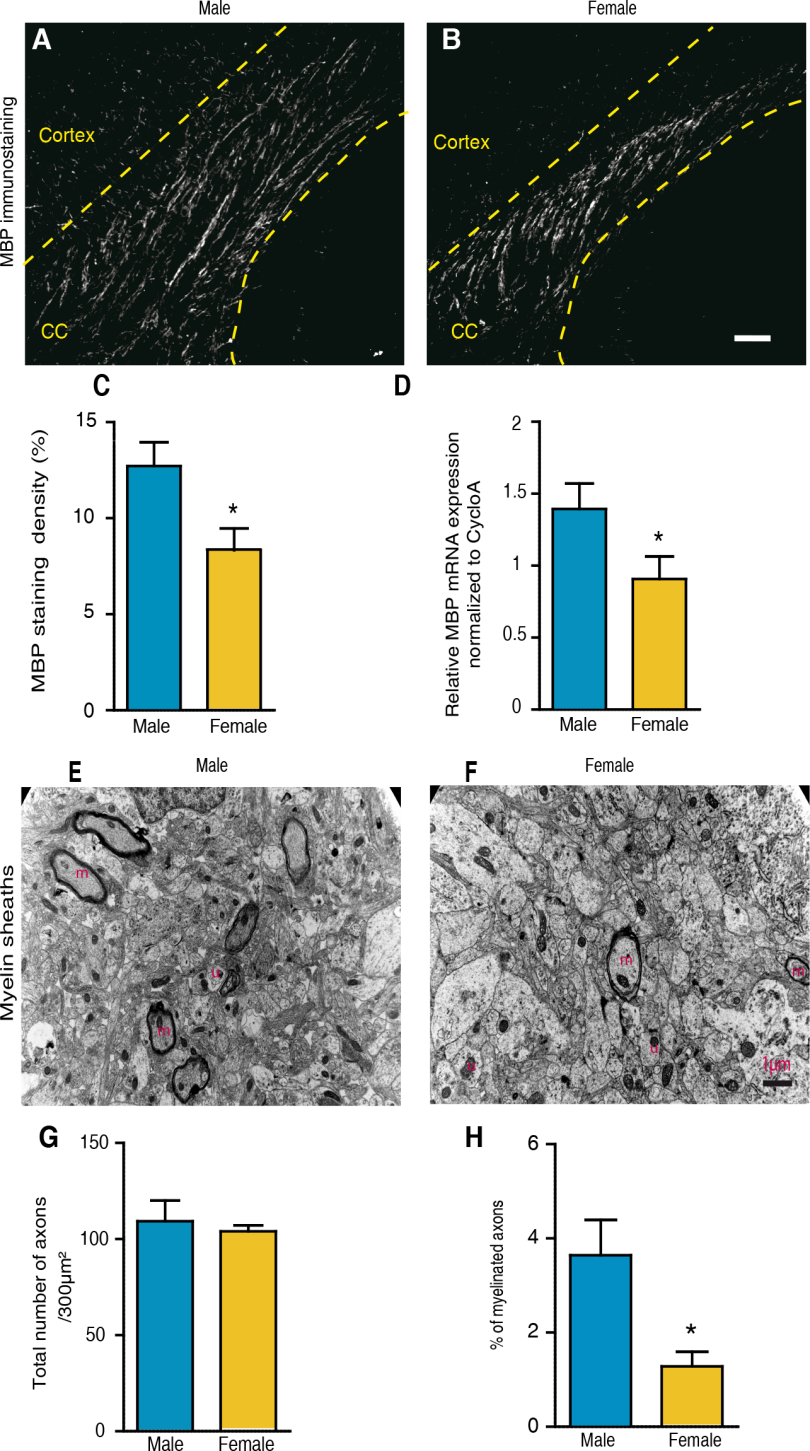
Corpus callosum myelin is sexually dimorphic at P10. (A and B) Immunostaining of myelin basic protein (MBP) on sagittal corpus callosum sections at P10 of (A) a male and (B) a female C57Bl/6 mouse. CC = corpus callosum. The dotted lines delimit the corpus callosum. (C) Quantification of the MBP immunostaining in P10 corpus callosum of male and female mice. For each animal, the MBP^+^ area was determined within 0.26 mm^2^ fields of splenium, center and genu using NIH image software and the mean area was calculated (n = 8). (D) MBP mRNA expression within the brain of males and female mice analyzed by qRT-PCR. The *cyclophilin A* gene was used for normalization (n = 3–4). (E and F) Electron micrographs of P10 corpus callosum of a (E) male and a (F) female WT mouse. Pictures show unmyelinated (u) and myelinated axons (m). (G-H) Analysis by electron microscopy of the total number of axons (G) and the percentage of myelinated axons (H) in corpus callosum at P10 (n = 3). Results are presented as means ± SEM. Significance was calculated using two-tailed Student’s t test (*p < 0.05 when compared to the corresponding male group). Scale bars: A and B = 20 μm; E and F = 1 μm.

The higher density of oligodendroglial cells at P10 might suggest an early sexual dimorphism of myelin. Sagittal brain sections from P10 male and female mouse brains were immunostained by using an antibody against MBP, a major component and established marker of CNS myelin [30]. Although myelin was still sparse at P10, there was nearly 35% more MBP-staining in corpus callosum of males when compared with females (Fig 2A–2C*)*. Consistent with the immunohistochemistry results, qRT-PCR analysis showed about 30% higher levels of MBP transcripts in the male brain when compared with females (Fig 2D).

Analysis by electron microscopy showed that only a small percentage of corpus callosum axons were myelinated at P10 (< 5%), but that there were more than twice as many myelinated fibers in males than in females. In contrast, the total number of axons did not differ between sexes (Fig 2E–2H). Thus, the density of oligodendrocytes, MBP expression and the number of myelinated axons already differ at P10 between sexes.

### Brain androgen levels and androgen receptor expression are higher in males than in females during the first ten postnatal days

**Fig 3:**
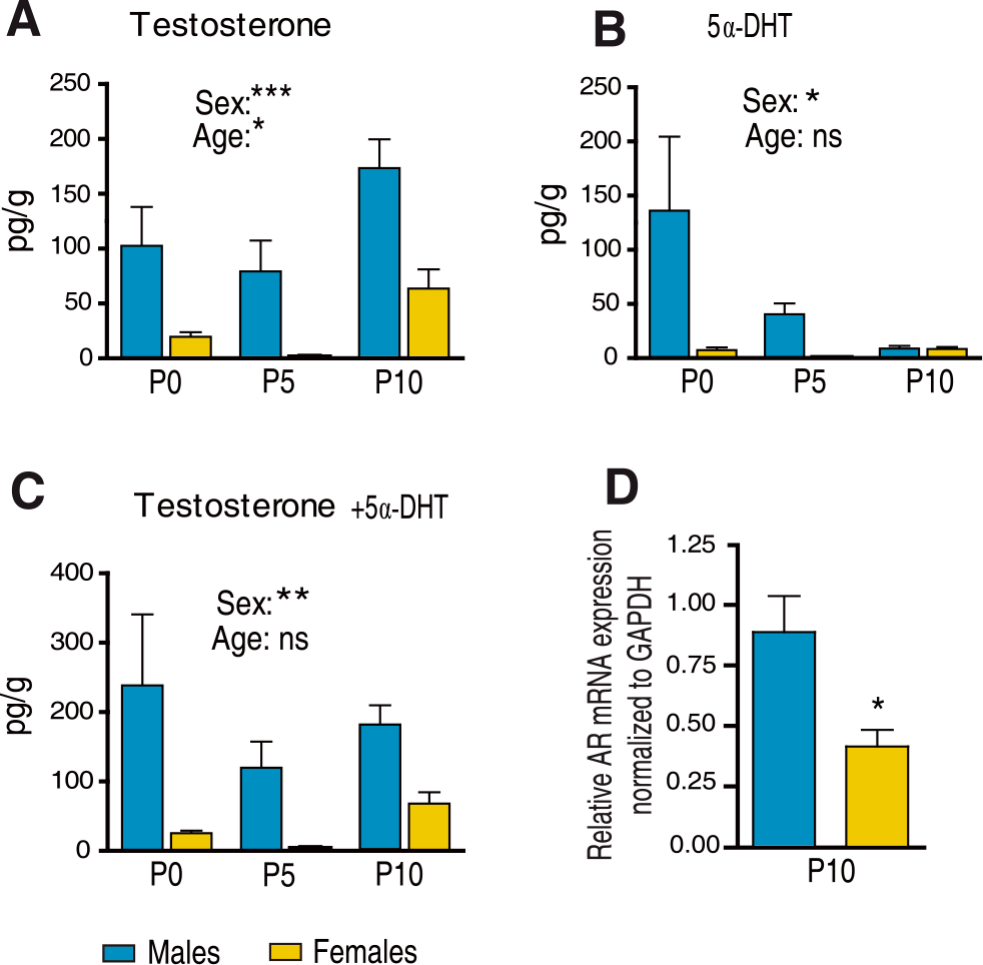
Brain levels of testosterone and 5α-dihydrotestosterone in males and females between P0 and P10 and androgen receptor expression. Comparison of brain levels of (A) testosterone, (B) 5α-dihydrotestosterone (5α-DHT) and (C) testosterone + 5α-DHT between male and female mice. Androgen levels were analyzed by GC-MS/MS at P0 (n = 16), P5 (n = 7–9) and P10 (n = 17–19). (D) Androgen receptor (AR) mRNA expression was analyzed within the brains of male and female mice by qRT-PCR. The GAPDH gene was used for normalization (n = 5). Results are presented as means ± SEM. Significance was calculated by two-way ANOVA with sex and age as factors. A significant effect of sex is indicated in the figures. (D) Comparison by two-tailed Student’s test (***p < 0.001; **p < 0.01; *p < 0.05 when compared to the corresponding male group).

To assess whether the observed sex difference in the density of oligodendrocytes and the extent of myelination at P10 may be dependent on the postnatal hormonal environment, we first analyzed brain steroid levels between P0 and P10 by GC-MS/MS. Levels of testosterone were higher in the male than in the female brain between P0 and P10 (Fig 3A). Their analysis by two-way ANOVA showed a significant effect of sex (F_(1,74)_ = 15.51, p < 0.0001) and age (F_(2,74)_ = 4.7, p = 0.012). The significant increase in testosterone at P10 in the male brain correlated to a marked drop of its immediate metabolite 5α-DHT (Fig 3B). At P0 and P5, brain levels of the more potent androgen 5α-DHT were higher in males, as compared to females (F_(1,75)_ = 3.72, p = 0.05).

The combined levels of testosterone and 5α-DHT, both ligands of AR, were significantly higher in the male than in the female brain at P0, P5 and P10 (Fig 3C). Analyzing their combined levels by two-way ANOVA showed a significant effect of sex (F_(1,77)_ = 10.6, p = 0.002). A more extensive profiling of brain steroids at P10 revealed that only levels of testosterone (p<0.001) and estradiol (p<0.05) were higher in males than in females, whereas the levels of 13 other steroids, including progesterone, were similar in both sexes (Table 1). The absence of sex differences in progesterone levels is interesting, as this neurosteroid also stimulates myelination during postnatal development [31, 32]. Higher brain levels of testosterone and 5α-DHT in males were concomitantly associated by significantly higher brain levels of AR mRNA, as determined by qRT-PCR (Fig 3D).

**Table 1:** Profiling of steroids in the male and female mouse brain at postnatal day 10.

Steroids	Males	Females	Detection limit
	(pg/g)	(pg/g)	(pg/g or pg/ml)
Testosterone	173 ± 26***	64 ± 17	1
5α-Dihydrotestosterone	9 ± 2	8 ± 2	2
Androstanediol	21 ± 3	19 ± 3	1
Androstenedione	66 ± 16	60 ± 14	2
Estradiol	5 ± 1.6*	1.5 ± 0.7	1
5α-Androstanedione	27 ± 3	19 ± 3	2
Pregnenolone	4812 ± 229	5543 ± 390	2
20α-dihydropregnenolone	192 ± 15	148 ± 30	1
Progesterone	56 ± 9	106 ± 41	20
5α-Dihydroprogesterone	670 ± 101	563 ±154	5
3β,5α-Tetrahydroprogesterone	6.1 ± 1.2	6.6 ± 2.2	0.5
20α-Dihydroprogesterone	205 ± 82	155 ± 46	10
Deoxycorticosterone	430 ± 100	716 ± 263	5
3α,5α-Tetrahydro deoxycorticosterone	18 ± 6	13 ± 3	2
Corticosterone	8295 ± 1519	9191 ± 2239	20

Profiling of steroids by gas chromatography coupled to tandem mass spectrometry (GC-MS/MS). Results are presented as means ± SEM (7< n > 19).

***p < 0.001;

*p = 0.05

### Sex differences in oligodendrocyte density and myelin at P10 are determined by postnatal AR signaling

**Fig 4:**
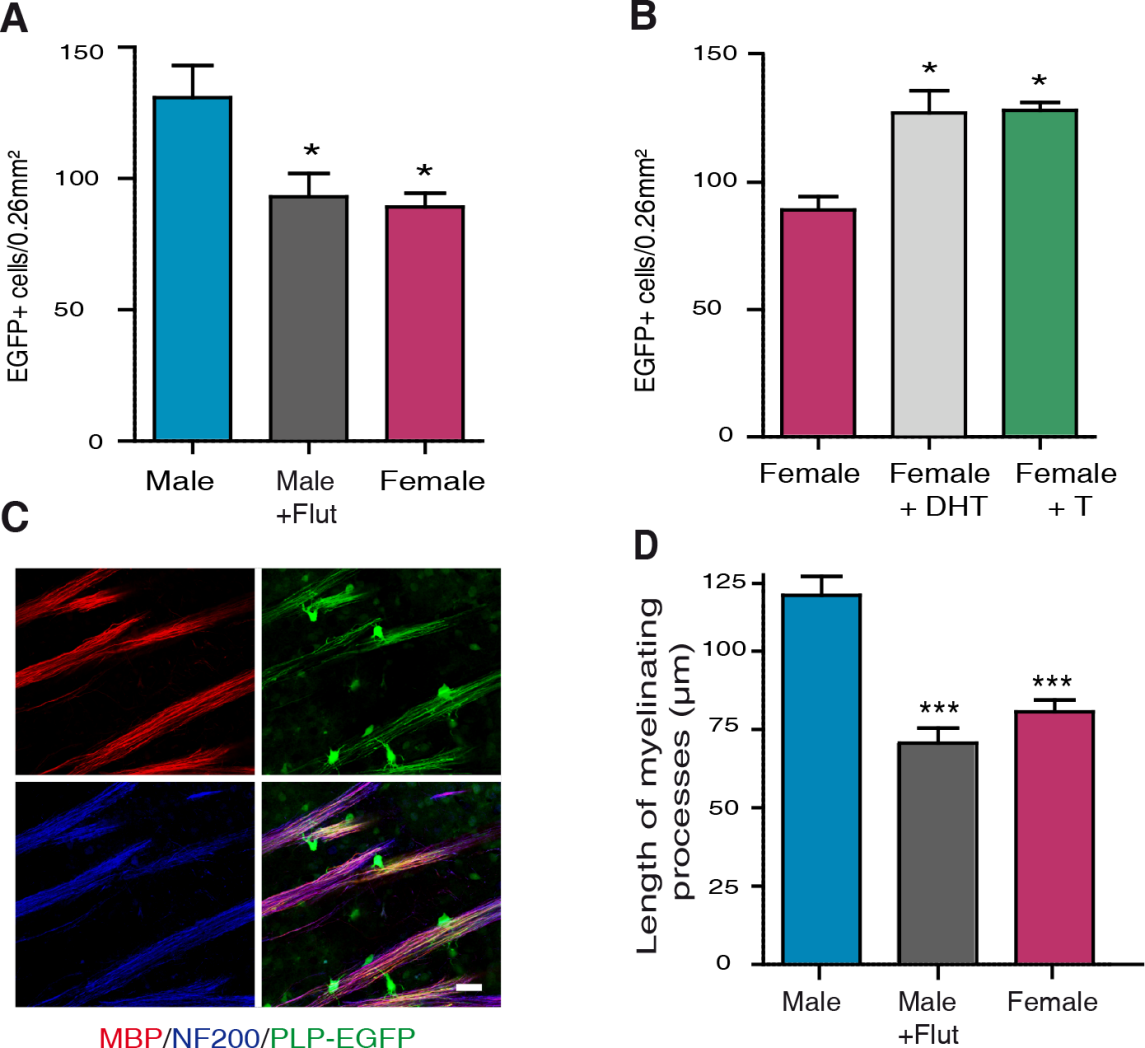
The role of androgens in determining sex differences in myelin at P10. (A) Comparison of the density of EGFP^+^ cells in corpus callosum of P10 PLP-EGFP male mice treated or not with the AR antagonist flutamide (Flut, 1 mg/kg), every two days between P0 and P10, and their control animals treated with sesame oil vehicle (n = 5). (B) Comparison of the density of EGFP^+^ cells in corpus callosum of P10 PLP-EGFP female mice treated with non-aromatizable 5α-dihydrotestosterone (DHT, 1 mg/kg), testosterone (T, 1 mg/kg) or with sesame oil vehicle, every two days between P0 and P10 (n = 5). (C) Immunolabeling of MBP^+^ myelin (red, top left) and NF200^+^ neurofilaments (blue, bottom left) on sagittal brain slices of PLP-EGFP^+^ P10 mice (green fluorescence, top right). The combination of all three markers (bottom right) shows oligodendrocytes and their processes (green) and myelinated axonal segments (purple). Photomicrographs were taken at the level of the junction between the striatum and the genu of corpus callosum. Scale bar = 20 μm. (D) Analysis of the myelinated axonal segments in P10 PLP-EGFP male mice treated or not with flutamide and female mice treated with sesame oil vehicle. (***p < 0.001; *p < 0.05 when compared to control males or females by one-way ANOVA followed by Newman-Keuls tests).

To investigate a link between AR signaling, the density of oligodendrocytes and the extent of myelination in corpus callosum at P10, male PLP-EGFP pups were subcutaneously injected every two days between P0 and P10 with 1 mg/kg of the selective AR antagonist flutamide. Blocking AR caused a 30% decrease in the density of EGFP^+^ oligodendroglial cells in the male corpus callosum at P10, which became similar to females (Fig 4A). Conversely, injecting female PLP-EGFP pups between P0 and P10 every two days with 1 mg/kg of testosterone or 5α-DHT, which is not aromatized into estrogens, increased the density of oligodendroglial cells in their corpus callosum to male-like levels (Fig 4B). To determine whether treatment with flutamide during the first 10 postnatal days also affected the organization of myelin, we measured at P10 the length of myelinated axon segments at the junction between the genu of the corpus callosum and the striatum, where they can be easily observed and quantified. Myelin segments were significantly longer in males when compared with females, but treatment of male pups with flutamide completely abolished this sex difference (Fig 4C and 4D).

**Fig 5:**
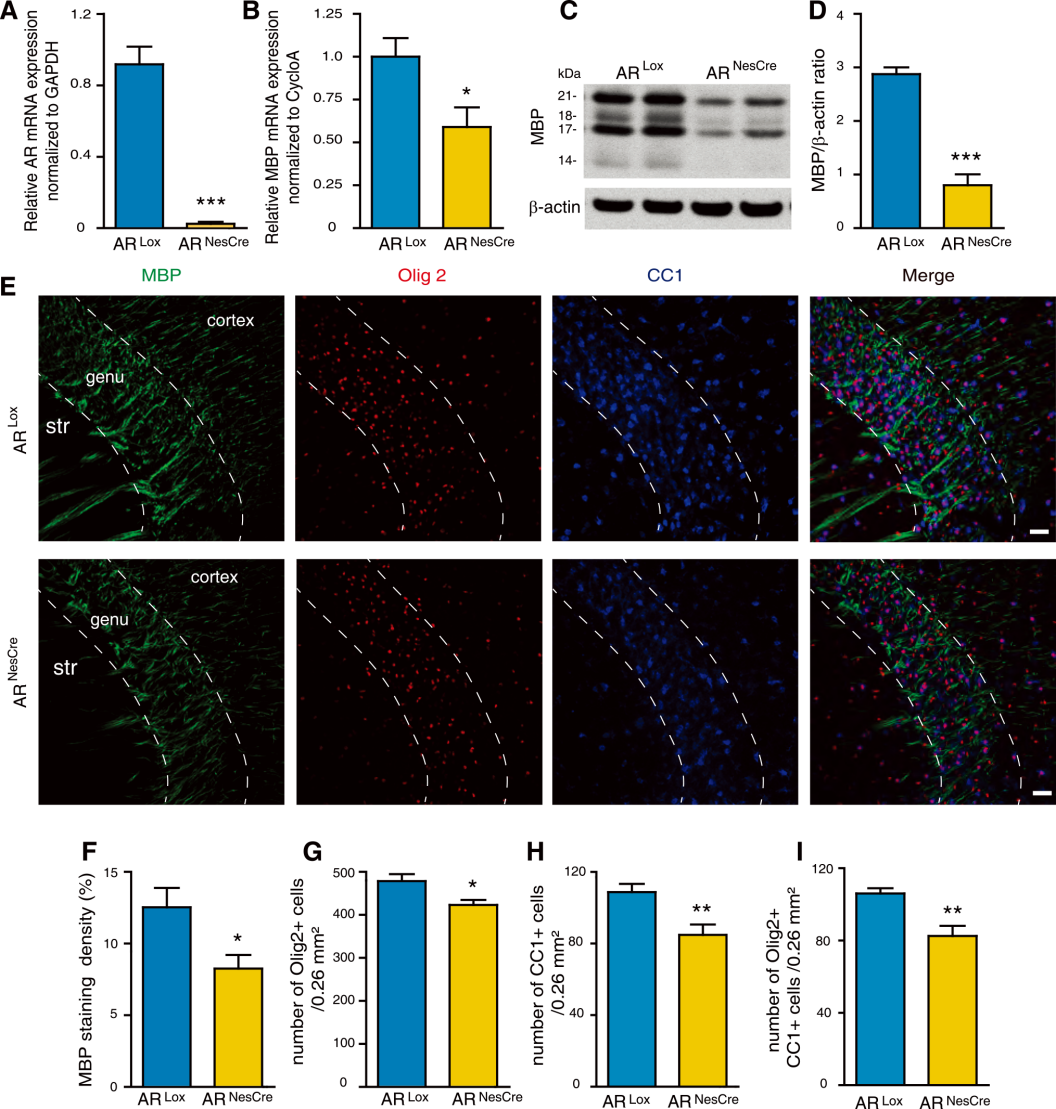
The role of brain androgen receptors in determining sex differences in myelin at P10. (A and B) Analysis by qRT-PCR of AR (A) and MBP (B) mRNA expressions in the brain at P10 of AR^NesCre^ male mice. Littermates carrying a floxed exon 1 of the *AR* gene (AR^Lox^) were used as controls (n = 4). GAPDH and *cyclophilin A* were used as normalization genes. (C and D) MBP protein levels, analyzed by Western blot and normalized to the endogenous β-actin protein, in the brain of AR^NesCre^ mice when compared to AR^Lox^ controls at P10 (n = 4). (E) Immunostaining of MBP (green), Olig2 (red), CC1 (mature oligodendrocytes, blue) and the merged triple immunostaining (yellow). Representative photomicrographs were taken at the level of the genu of the corpus callosum (str = striatum). Scale bar = 100 μm. (F-I) Within the P10 corpus callosum, (F) quantification of the MBP^+^ area in a 0.26 mm^2^ field and counting of (G) Olig2^+^ oligodendroglial cells, (H) CC1^+^ mature oligodendrocytes and (I) Olig2/CC1 double positive cells in AR^NesCre^ mice compared with AR^Lox^ littermates (n = 6). Results are presented as means ± SEM. For each animal, the mean value of the corpus callosum was calculated from the splenium, the center and the genu. Significance was calculated using two-tailed Student’s t test (***p < 0.001; *p < 0.05 when compared to the corresponding control AR^Lox^ males).

Systemic treatment with flutamide or 5α-DHT affects AR signaling in the entire body. To assess the role of cerebral AR in the development of postnatal sex differences in corpus callosum myelin, we deleted AR in neural cells using the Cre/Lox system. A mouse line carrying a floxed exon 1 of the AR gene, located on chromosome X, was used. In this line, Cre recombination results in the excision of the transcription start site and deletion of the N-terminal domain of AR [33]. Female AR^Lox^ mice were crossed with male mice expressing the Cre recombinase under the control of the promoter and the CNS-specific enhancer of rat nestin [34]. This NesCre mouse line is characterized by a very efficient and selective Cre-mediated recombination [35, 36]. In the resulting male AR^NesCre^ mice, AR expression was deleted in CNS neurons, astrocytes and oligodendrocytes, but not in microglial cells. AR^Lox^ males were used as controls. The specific knockout of AR expression in the brain of AR^NesCre^ males was verified by qPCR analysis (Fig 5A). AR mRNA expression was not affected in muscle and testis.

The genetic deletion of AR in CNS neurons and macroglial cells resulted in markedly reduced MBP expression in the brain at P10. Levels of MBP mRNA transcripts and protein isoforms were decreased by almost 40% and 70%, respectively (Fig 5B–5D). Immunofluorescence analysis of the corpus callosum at P10 showed a significant reduction in MBP staining density, as well as in the number of Olig2^+^ cells and mature oligodendrocytes (CC1^+^ and CC1^+^/Olig2^+^ cells) in AR^NesCre^ males when compared with AR^Lox^ males (Fig 5E–5I).

Both pharmacological and genetic inhibition of AR thus showed that postnatal androgens, via their receptor, are involved in the sexual dimorphism that affects the density of oligodendrocytes and the extent of myelination at P10.

### Persistent effects of early postnatal androgens on adult sex differences in myelin

To assess whether exposure to androgens during the first ten postnatal days has a long-lasting impact on the sexual phenotype of corpus callosum myelin, we first used organotypic cultures of cerebellar slices prepared from P10 male and female PLP-EGFP pups [37]. At this stage, the myelination of axons only starts in cerebellum [31, 38]. Importantly, the cerebellar tissue was exposed prior to culture to higher levels of endogenous testosterone and 5α-DHT in males when compared with females. The cerebellar slices were then cultured for 2 weeks to allow axons to become fully myelinated.

**Fig 6:**
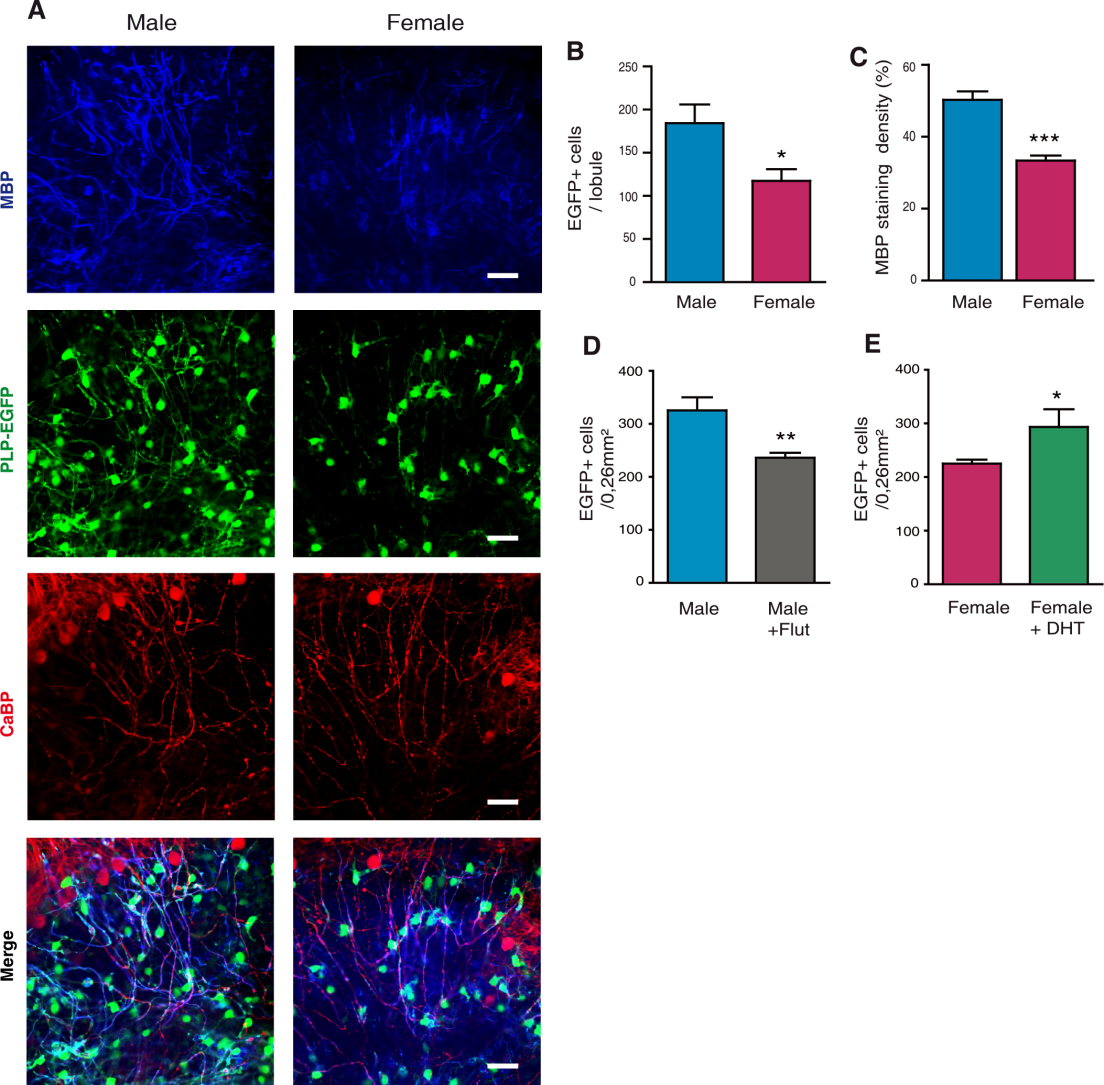
Androgen-dependent sex differences in myelin are maintained over time: *In vivo* and *in vitro* studies. (A) Immunostaining of MBP^+^ myelin (blue) and EGFP^+^ oligodendroglial cells (green) in cerebellar slices taken from PLP-EGFP male and female mice at P10 and maintained in culture for two weeks in the absence of androgens. The Purkinje neuron marker Calbindin (CaBP) (red) was used to track nerve cells. The merged triple immunostaining (bottom) documented the higher myelination of the male cerebellar slices. (B and C) Analysis of the density of EGFP^+^ oligodendroglial cells (B) and of the MBP^+^ area (C) within the male and female cerebellar slices. (n = 5 animals, three slices and three regions from each slice were analyzed per animal). (D) Injecting male PLP-EGFP mice every two days between P0 and P10 with the AR antagonist flutamide (Flut, 1 mg/kg) and control mice with sesame oil vehicle. Analysis of EGFP^+^ oligodendroglial cells was performed at 3 months of age (n = 5). (E) Injecting female PLP-EGFP mice every two days between P0 and P10 with 5α-dihydrotestosterone (DHT, 1 mg/kg) and control mice with sesame oil vehicle. Analysis of EGFP^+^ oligodendroglial cells was performed at 3 months of age (n = 5). Results are presented as means ± SEM. Significance was calculated using two-tailed Student’s t test (***p < 0.001; **p < 0.01 *p < 0.05 when compared to the corresponding control).

Although the culture medium contained 25% horse serum, levels of androgens in the culture medium were below the limit of detection by GC-MS/MS (1 pg/ml for testosterone and 2 pg/ml for 5α-DHT, see Table 1). Therefore, myelination proceeded to completion in an androgen-deprived medium. Consistent with a masculinizing effect of androgens on the process of myelination prior to P10, the density of EGFP^+^ oligodendroglial cells in the cerebellar lobules was 36% higher in slices prepared from male pups than in those prepared from female pups (Fig 6A and 6B). Furthermore, MBP staining density was about 30% higher in the male slices (Fig 6A and 6C). These observations are consistent with a persistent effect of the postnatal androgen environment on myelin.

To determine whether postnatal androgen-dependent sex differences in myelin persist into adulthood, we treated again male PLP-EGFP pups with flutamide and female pups with 5α-DHT between P0 and P10 (1 mg/kg every two days). We then counted oligodendrocyte cells in their corpus callosum at the age of 3 months. As for P10, postnatal treatment with flutamide decreased the density of oligodendroglial cells in the adult male corpus callosum by 30% (Fig 6D). Conversely, treating female PLP-EGFP pups between P0 and P10 every two days with 5α-DHT increased the density of oligodendroglial cells in the adult corpus callosum by about 20% (Fig 6E). Thus, in spite of the constantly very low levels of endogenous androgens in females, their exposure to exogenous 5α-DHT during the first 10 postnatal days was sufficient to induce a male-like density of oligodendrocytes in their corpus callosum. Both *in vitro* and *in vivo* experiments thus documented persistent influences of postnatal androgens on myelin.

### Adult male AR^NesCre^ mice exhibit a female-like phenotype of myelin

**Fig 7:**
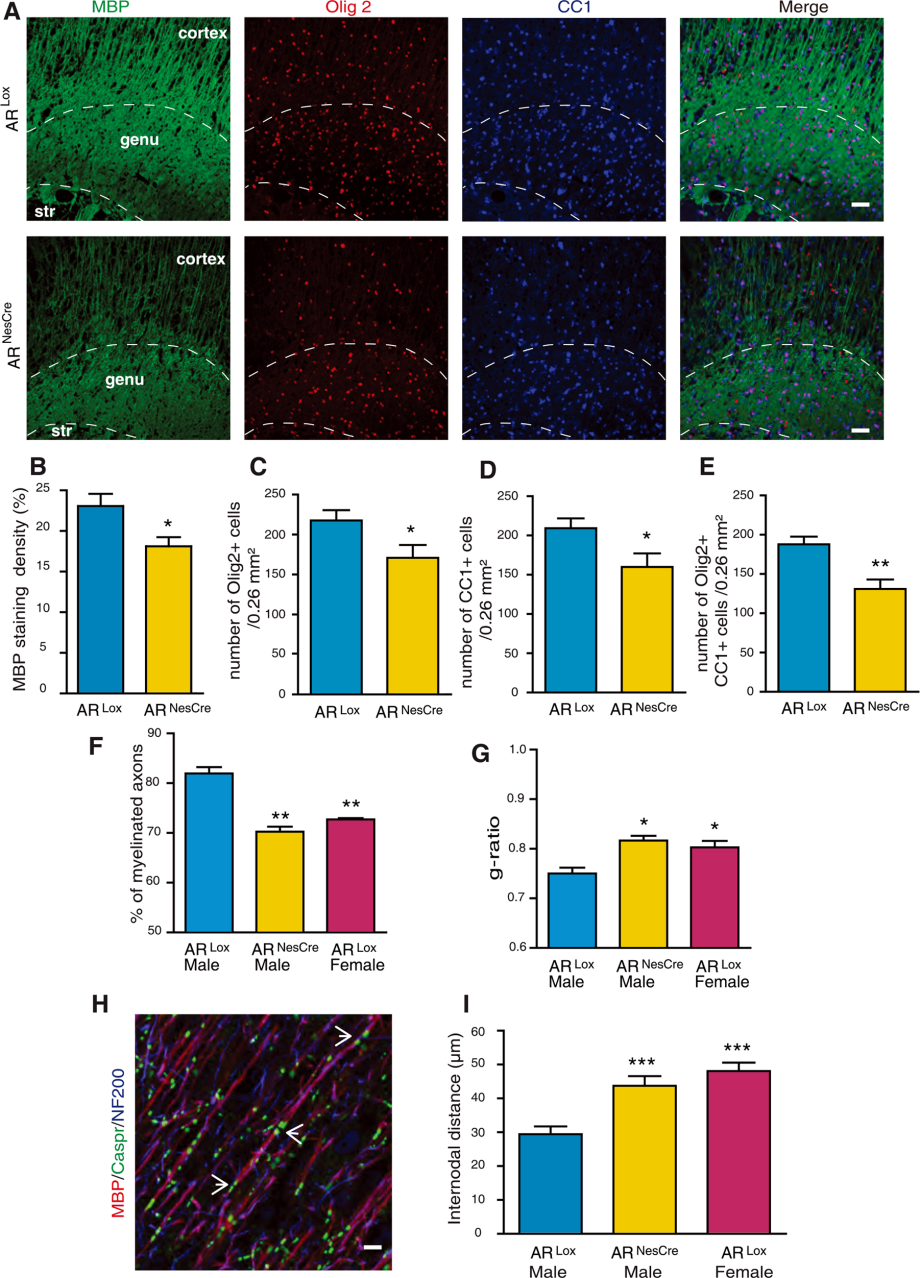
A female-like phenotype of myelin in adult AR^NesCre^ males. (A) Immunostaining of MBP (green), Olig2 (red), CC1 (mature oligodendrocytes, blue) and the merged triple immunostaining. Representative photomicrographs were taken at the level of the genu of the corpus callosum (str = striatum) of 3 months old AR^Lox^ control and AR^NesCre^ males. Scale bar = 100 μm. (B-E) Within the corpus callosum of 3 months old AR^Lox^ and AR^NesCre^ males, quantification of the MBP^+^ area (B) and counting of Olig2^+^ oligodendroglial cells (C), CC1^+^ mature oligodendrocytes (D) and Olig2/CC1 double positive cells (E), (n = 6). (F and G) Analysis by electron microscopy of the percentage of myelinated axons (F) and g-ratios of myelinated axons (G) in corpus callosum of adult AR^Lox^ males and females and AR^NesCre^ males (n = 3). (H) Immunostaining of the paranodal junction marker, Caspr (contactin associated protein, green, pointed by white arrows), MBP^+^ myelin (red) and Neurofilament (NF200, blue) in sagittal sections of the adult male mouse cerebral cortex. Scale bar = 5 μm. (I) Comparison of internodal distances in cerebral cortex between adult AR^Lox^ males, AR^Lox^ females and AR^NesCre^ males (n = 5). Results are presented as means ± SEM. Significance was calculated using two-tailed Student’s t test for comparisons between two groups and Newman-Keuls tests after one-way ANOVA for comparisons between three groups (***p < 0.001; **p < 0.01; *p < 0.05 when compared to AR^Lox^ males).

Conditional deletion of AR in the brain had a major impact on the density of oligodendroglial cells and the extent of myelination in the male corpus callosum at P10. Moreover, postnatal androgens exerted long-lasting masculinizing effects on myelin, extending into adulthood. Adult AR^NesCre^ male mice were thus expected to exhibit a female-like phenotype of myelin with a reduced density of oligodendroglial cells and decreased MBP immunostaining. Indeed, immunohistochemical analysis of the corpus callosum by fluorescence microscopy revealed that at the age of 3 months, the densities of Olig2^+^ oligodendroglial cells, CC1^+^ mature oligodendrocytes and Olig2/CC1 co-expressing oligodendrocytes were reduced by 20 to 30% in AR^NesCre^ male mice when compared with AR^Lox^ controls, thus becoming similar to females (Fig 7A and 7C–7E). On the other hand, MBP immunostaining was decreased by 20% in corpus callosum of AR^NesCre^ males (Fig 7A and 7B).

Analysis by electron microscopy showed that the percentage of myelinated axons in corpus callosum was decreased in AR^NesCre^ males by 15% when compared to control AR^Lox^ males, but was similar to AR^Lox^ females (Fig 7F). However, the total number of callosal axons was not affected by AR deletion in the brain. The mean g-ratio of myelinated callosal axons was significantly higher in AR^NesCre^ males than in controls and similar in AR^NesCre^ males and in AR^Lox^ females (Fig 7G). The thickness of the axons is approximately 0.89 +/- 0.07 μm in AR^NesCre^ and 0.90 +/- 0.08 μm in AR^Lox^ whereas that of the whole fiber is approximately 1,09 +/- 0,06 μm in AR^NesCre^ and 1.21 +/- 0.10 μm in AR^Lox^ males. This suggests that myelin sheaths are thinner in males after AR deletion, becoming comparable to females.

Each myelinated axon segment between two nodes of Ranvier, named internode, is formed by a single oligodendrocyte process. Importantly, oligodendrocytes can myelinate several internodes. Thus, an increase in the density of oligodendroglial cells may result in a decreased number of internodes formed per oligodendrocyte, or alternatively in shorter internodes [39]. The latter is what we observed by measuring internodal distances between two paranodal regions in cerebral cortex, where extended parts of myelinated axons can be observed in a same plane. The length of internodes of myelinated axons was measured after triple immunolabeling of contactin-associated protein (Caspr), a glycoprotein present in the paranodal region, of MBP and of neurofilaments (NF200) (Fig 7H). Internodal distances in the cortex of AR^Lox^ males were about 40% shorter than in AR^NesCre^ males, and they were comparable to normal females (Fig 7I). Thus, AR^NesCre^ males exhibited a female-like phenotype of myelin, characterized by thinner myelin sheaths and longer internodes.

### Adult male AR^Tfm^ mice exhibit a female-like phenotype of myelin

The conditional deletion of AR in the brain demonstrated its importance in the masculinization of myelin. To corroborate this finding, myelin was also examined in adult male mice carrying the naturally occurring AR testicular feminization mutation (AR^Tfm^). In these mice, a frame shift mutation in exon 1 of the *AR* gene results in a nonfunctional receptor in the entire body [40, 41].

**Fig 8:**
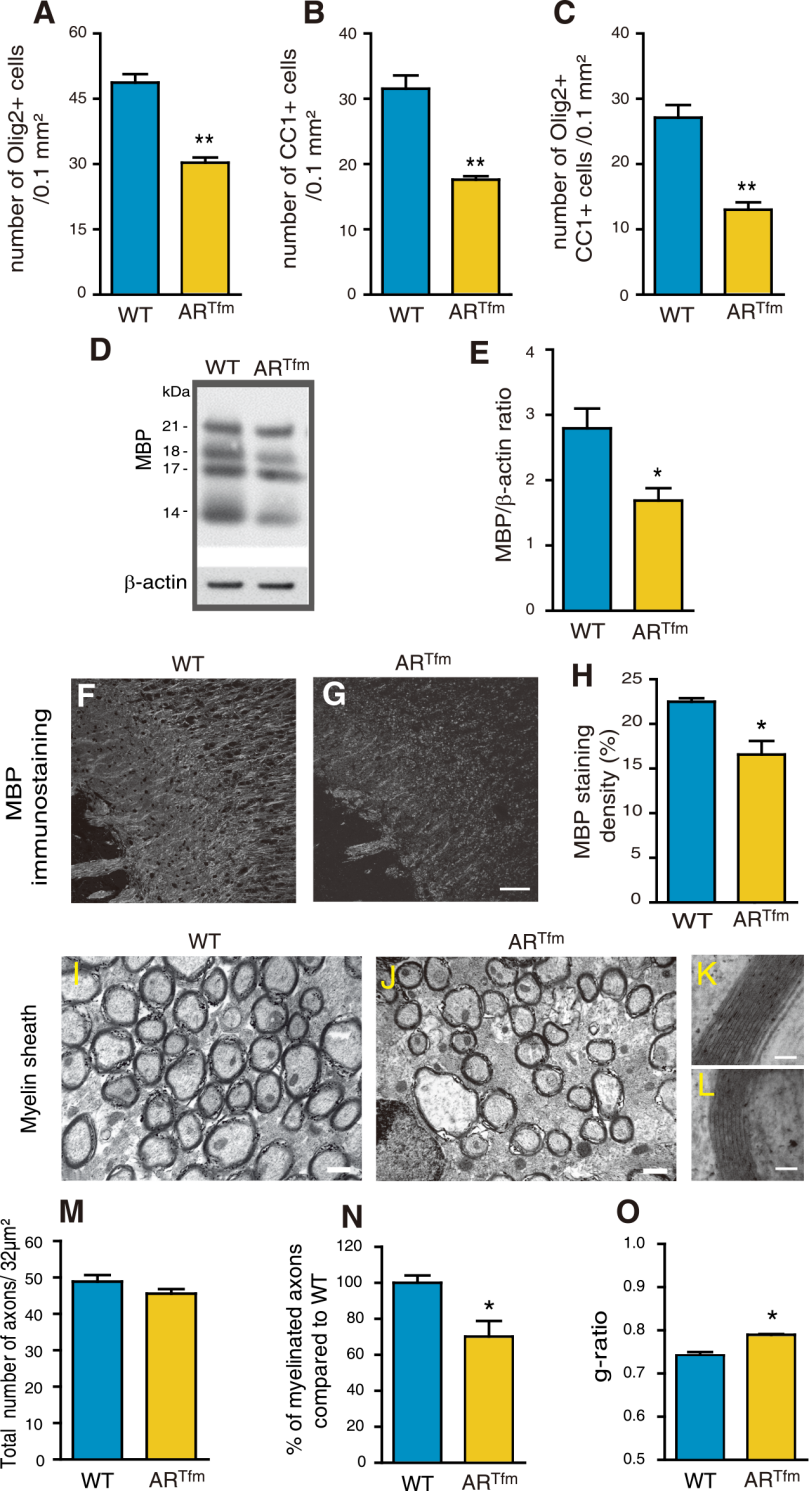
A female-like phenotype of myelin in adult AR^Tfm^ males. Within the corpus callosum of 3 months old wild type males (WT) and males carrying the testicular feminization mutation (AR^Tfm^) (n = 6), quantification of the density of Olig2^+^ oligodendroglial cells (A), CC1^+^ mature oligodendrocytes (B) and Olig2/CC1 double positive cells (C). (D and E) Representative Western Blot analysis and quantification of all MBP isoforms, normalized to the endogenous β-actin protein, in brain of WT and AR^Tfm^ males (n = 4). (F and G) MBP immunostaining of corpus callosum in sagittal brain sections from WT (F) or AR^Tfm^ males (G). Scale bar = 100 μm. (H) Quantification of the MBP positive area density in a 0.26 mm^2^ field in the corpus callosum of WT and AR^Tfm^ mice (n = 6). For each animal, the mean value of the corpus callosum was calculated from splenium, center and genu. (I-L) Representative electron micrographs of nerve fibers in corpus callosum of adult WT (I) and AR^Tfm^ (J) male mice. Scale bar = 0.5 μm. High magnification electron micrographs of a representative axon and its myelin sheath in WT and AR^Tfm^ males, (K and L) respectively. Scale bar = 50 nm. (M-O) Analysis by electron microscopy of the total number of axons (M), the percentage of myelinated axons (N) and the g-ratios (O) in corpus callosum of WT and AR^Tfm^ males (n = 3). Results are presented as means ± SEM. Significance was calculated using two-tailed Student’s tests (**p < 0.01; *p < 0.05; when compared to the corresponding control WT males).

The reduction in the densities of Olig2^+^ oligodendroglial cells, CC1^+^ oligodendrocytes and Olig2/CC1 co-expressing oligodendrocytes in the corpus callosum of 3 months AR^Tfm^ males was even more marked than for AR^NesCre^ males, reaching 40 to 50% when compared with wild-type (Fig 8A–8C). Consistently, the amount of MBP analyzed by Western blot (Fig 8D and 8E) and MBP immunostaining in corpus callosum (Fig 8F–8H) were significantly lower in these AR^Tfm^ males when compared with wild-types. Particularly marked was the reduction in myelinated axons within the corpus callosum of AR^Tfm^ males and the thinner myelin sheaths observed by electron microscopy (Fig 8I–8O). The thinner myelin in AR^Tfm^ males was reflected by a significant increase in the mean g-ratio (Fig 8O). However, the density of callosal axons did not differ between wild types and AR^Tfm^ males (Fig 8M). Taken together, the myelin phenotype of AR^Tfm^ mice resembled the one of AR^NesCre^ mice, but differences with the controls appeared to be more marked.

## Discussion

The main questions addressed throughout this study were whether sex difference in myelin emerge early during mouse brain development, and whether they are determined by postnatal androgens. We found that the sexual dimorphism of myelin and oligodendrocytes arises between P5 and P10 and involves long-lasting effects of androgen signaling via AR. At this developmental stage, only a small percentage of corpus callosum axons are myelinated and they show very little variation in diameter [42].

The PLP-EGFP mouse, in which the PLP promoter drives high and selective EGFP expression in the oligodendroglial lineage at early stages of their development [23], allowed a refined analysis of the developmental emergence of sex differences in oligodendrocytes and myelin. We observed that the male corpus callosum contains more Olig2^+^ glial cell progenitors already at P5, when the density of more mature EGFP^+^ oligodendroglial cells still does not differ between sexes. At this early stage, Olig2 is still expressed in a large number of gliogenic progenitors, and one of its early functions is the repression of neuronal differentiation. These early Olig2^+^ glial progenitors can still give rise to either oligodendrocytes or astrocytes, and it is only later that Olig2 becomes restricted to oligodendroglial cells and is required for their maturation [26, 27]. However, at P10, the densities of Olig2^+^/EGFP^+^ oligodendroglial cells and Olig2^+^/CC1^+^ mature oligodendrocytes were significantly higher in the corpus callosum of males when compared with females. Consistently, MBP immunostaining was slightly denser and the percentage of myelinated axons was higher in males. These results contrast with the current view that sex differences in the number of myelinated callosal axons only appear around puberty, when the size of the corpus callosum becomes larger in males [43].

### Higher levels of testosterone and 5α-DHT in the postnatal male brain and their role in the masculinization of myelin

Analyses by GC-MS/MS revealed that between P0 and P10, at the time when the process of myelination begins and sex differences in myelin emerge, brain levels of testosterone and 5α-DHT are significantly higher in males than in females. This is after the masculinizing surge of testosterone, which takes place around birth in males and only lasts for a few hours [13, 44, 45]. Interestingly, an earlier biochemical work in rats already reported higher levels of ligand-occupied nuclear AR in male pups when compared with females during the postnatal period [46]. This sex difference in AR ligand availability is consistent with a masculinizing action of postnatal androgens during developmental myelination.

A second important observation was that brain levels of 5α-DHT are markedly higher in males at P0 and P5, but drop to low female-like levels at P10. This developmental period corresponds to the transient expression in the brain of the type 2 isoform of the 5α-reductase, which has a higher affinity for testosterone than the type 1 isoform and is normally expressed in peripheral androgen-target tissues such as the prostate [47]. Although both testosterone and 5α-DHT act through a single receptor, 5α-DHT is a more potent agonist ligand of AR. The conversion of testosterone to 5α-DHT thus amplifies the androgenic signal [48]. Moreover, in contrast to testosterone, 5α-DHT cannot be converted to estrogens and its formation thus selects the AR signaling pathway. The transient elevation in brain levels of 5α-DHT in males is likely to play a role in the masculinization of myelin. Indeed, the density of EGFP^+^ oligodendroglial cells in corpus callosum and the length of myelinated processes below the genu were reduced in the P10 male brain by the AR antagonist flutamide, which also inhibits expression of the 5α-reductase type 2 [47, 49]. Conversely, giving 5α-DHT to female pups between P0 and P10 resulted in a male-like density of oligodendroglial cells at P10. These two observations also provided a first line of evidence for a key role of AR signaling in the sexual differentiation of myelin. Consistently, AR mRNA expression was higher in the postnatal male brain when compared with females.

### The role of postnatal AR signaling in sexual differentiation of brain myelin

We provide evidence for a key role of the brain AR in the masculinization of myelin by using AR^NesCre^ mice with selective excision of AR in neural cells of the CNS. Under the control of the nestin promoter, the Cre recombinase is expressed in neural precursor cells as early as embryonic day 10 [34]. Cre-dependent excision of the floxed exon 1 of the *AR* gene resulted in complete AR invalidation in the brain. This genetic tool allowed us also to avoid the confounding effects of systemic hormonal changes caused by injections of flutamide or 5α-DHT.

At P10, both mRNA and protein levels of MBP were markedly reduced in the brain of AR^NesCre^ males when compared with control AR^Lox^ males. Within the corpus callosum, MBP immunostaining, the densities of Olig2^+^ cells and of mature oligodendrocytes expressing CC1 or coexpressing CC1 and Olig2, were also significantly reduced in AR^NesCre^ males. These observations demonstrate that sex differences in myelin observed at P10 are dependent on the presence of a functional AR in the male brain.

We then addressed the important question of a hormonal imprinting of developmental myelination by early postnatal androgens. We first used organotypic cultures of cerebellar slices prepared from P10 male and female PLP-EGFP mice. Remarkably, although cerebellar slices were cultured during 2 weeks in the absence of detectable levels of androgens, the myelin formed *in vitro* differed between sexes, with a higher density of EGFP^+^ oligodendroglial cells and of MBP^+^ staining density in males. This observation was consistent with a persistent effect of the postnatal androgen environment on myelin prior to culture.

We then demonstrated that postnatal androgen-dependent sex differences in myelin persist into adulthood. Treatment of male pups with flutamide between P0 and P10 reduced the density of EGFP^+^ cells in the adult corpus callosum to female-like levels. Conversely, treatment of female pups with 5α-DHT between P0 and P10 significantly increased the density of callosal EGFP^+^ cells in adults. It is important to emphasize that females treated during their first 10 postnatal days with 5α-DHT were no longer exposed to significant levels of androgens afterwards. Thus, postnatal androgens have persistent effects on myelin, independent on the later hormone environment.

### The phenotype of adult myelin in the absence of AR

Because of the persistent and AR-dependent effects of postnatal androgens on myelin, a reduced density of oligodendrocytes and MBP expression could be expected in the corpus callosum of adult mice lacking functional AR. This was indeed observed when comparing 3 months old AR^NesCre^ males with AR^Lox^ males. Moreover, analysis of corpus callosum axons at the electron microscopic level revealed a reduction in the percentage of myelinated axons and in the thickness of the myelin sheaths, reflected by an increased g-ratio in AR^Lox^ males.

Oligodendrocytes myelinate multiple internodes on different axons. Thus, when their number is reduced, a single oligodendrocyte can be expected to myelinate more axonal segments or to form longer segments of myelin (internodes). In cerebral cortex, where internodes can be measured accurately, their mean length was significantly increased in AR^NesCre^ males when compared with AR^Lox^ males.

Adjusting internode length has been identified as a means of regulating conduction velocity [50]. However, the underlying determinants remain poorly understood. Thus, variations in internode length have been proposed to result from neuronal signals during development or to reflect neuron-independent intrinsic properties of oligodendrocytes [51]. A recent study has shown that in organotypic cultures of cerebral cortex slices, a reduced number of oligodendroglial cells resulted in longer internodes [52]. Our results uncover a novel role of neural AR signaling in determining internode length.

Longer internodes in AR^NesCre^ males and in females, when compared with control AR^Lox^ males, should result in faster conduction velocities. However, AR^Lox^ males have thicker myelin sheaths, which reduce capacitance along internodes, thus allowing a faster propagation of action potentials. We do not know whether the thicker myelin sheaths compensate for the shorter internodes in males, nor how the sex-specific characteristics of myelin affect information processing in the brain. The analysis of conduction velocities and compound action potentials of fast conducting myelinated axons in mouse corpus callosum has so far not allowed identifying significant differences between males and females [53]. However, these measures reflect mean responses of large numbers of electrically stimulated nerve fibers. Thus, further studies are necessary to shed more light on this matter.

The myelin phenotype in the corpus callosum of AR^Tfm^ males, with a nonfunctional AR in all tissues, strongly resembled the one observed in AR^NesCre^ males, including a lower density of oligodendroglial cells, a decreased MBP expression, a smaller percentage of myelinated axons and thinner myelin sheaths with higher g-ratio values. Although in AR^Tfm^ mice, the absence of functional AR in all tissues is accompanied by endocrine abnormalities, this model has been widely used to probe the role of AR in shaping brain and behavior in rodents [54]. Most important, related mutations of the *AR* gene in humans, also known as complete androgen insensitivity syndrome (CAIS), suggest that functional AR are required to masculinize the human brain [55]. Indeed, a recent study using diffusion tensor imaging has shown that individuals with CAIS show female-typical characteristic of white matter microstructure [56].

### Persistent effects on myelin of postnatal AR signaling versus myelin plasticity

Although influences of postnatal androgens on myelin are long lasting, they may not be entirely irreversible. In the adult brain, myelin indeed shows structural plasticity and myelin remodeling has been proposed to participate in cognitive processes [57, 58]. Although the majority of oligodendrocytes are generated during the first postnatal weeks in mice, Oligodendrocyte Progenitors (OP) remain present in the adult brain, where they continue to differentiate into oligodendrocytes and form new myelin sheaths [59, 60]. The slow remodeling of myelin, which takes place throughout life and involves adult OP, could also be affected by the presence or absence of androgens. Thus, long-term castration of adult male mice resulted after 3 months in a more female-like phenotype of myelin characterized by fewer oligodendrocytes [61].

The role of androgens indeed goes beyond the sexual differentiation of myelin during development, as both testosterone and AR also play a key role in the regeneration of adult myelin. We have recently shown that testosterone stimulates the formation of new myelin in a mouse model of severe and chronic demyelination. In this study, we also identified the neural AR as a key target for the remyelinating actions of testosterone [36]. Of note, after severe cuprizone-induced demyelination of the corpus callosum, testosterone treatment stimulated the formation of new myelin with a male-like phenotype in both sexes [36]. Moreover, our recent study shows that after the acute demyelination of axons in the ventral white matter of the spinal cord, testosterone and a functional AR in the CNS are required for the spontaneous regeneration of myelin by oligodendrocytes [62].

### Sexual differentiation of myelin: Significance for demyelinating diseases

Despite a greater susceptibility to multiple sclerosis (MS), women have a better prognosis with respect to disability progression than men [63] [64] [65]. Sex-related differences in experimental autoimmune encephalomyelitis (EAE), an accepted model of MS, are in line with these clinical observations [66]. As an explanation to this disparity, other than distinct immune mechanisms in both sexes, hormone-dependent mechanisms of neuronal resilience have been proposed [67]. The organization of the myelin sheaths may indeed impact their integrity and vulnerability to immune attacks [68, 69]. Thus, the long-term developmental effects of androgens on myelin assembly, observed in the present work, may contribute to sex differences in the maintenance and regeneration of the myelin sheaths and their vulnerability to immune attacks. Therefore, the present observations in addition to our previous results, uncovering the efficacy of androgens as remyelinating agents [36, 62], provide a new conceptual framework for myelination and remyelination processes, with potential implications for demyelinating diseases such as multiple sclerosis.

## Matherials and methods

### Animals

All procedures were performed according to the European Communities Council Directive (86/806/EEC) for the care and use of laboratory animals. All mice except the AR^Tfm^ (testicular feminization mutation, see below) were bred in our animal facility under a 12 hours dark/light cycle with food and water *ad libitum*. All mice were healthy with no obvious behavioral phenotypes, and none of the experimental mice was immune compromised.

Mouse lines used in this study are the following: newborn (P0), 5 days old (P5) or 10 days old (P10) C57Bl/6 wild type mice and mice expressing the enhanced green fluorescent protein under the control of the proteolipid protein gene promoter PLP-EGFP [23] at P5, P10 and adulthood. Mice of either sex were used and were randomly allocated to experimental groups.

AR^Tfm^ male mice, which carry a naturally inactivating mutation of the AR, were obtained from the French Atomic Energy Commission [41].

We also generated mice lacking the androgen receptor in neural cells, using the Cre/Lox system. A mouse line carrying a floxed exon 1 of the *AR* gene, located on chromosome X, was provided by CIE-CERBM of IGBMC (Pr. Pierre Chambon) [70]. In this line, Cre recombination results in the excision of the transcription start site and deletion of the N-terminal domain of AR [33]. Female AR^Lox^ mice were crossed with male mice expressing the Cre recombinase under the control of the promoter and the CNS-specific enhancer of rat nestin [34]. In experiments involving these mice, AR^Lox^ male littermates were used as controls to maintain the same genetic background.

### In vivo study

In case of steroid injections, testosterone (T), 5α-dihydrotestosterone (5α-DHT) or flutamide were dissolved in sesame oil at an optimal concentration of 1 mg/ml. PLP-EGFP pups were subcutaneously injected with 20 μl of these solutions every 2 days from P0 till P10 whereas control animals were injected with sesame oil. Mice were sacrificed at P10 or at 3 months of age. Mice used for immunofluorescence studies were intracardially perfused with 4% paraformaldehyde (PFA) in phosphate buffer. Brains were dissected and post-fixed in the same solution for at least two days. 50μm thick sagittal slices were cut using a vibratome (Leica).

### Organotypic slice cultures

Slice cultures were done as described earlier [32, 36]. Briefly, after decapitation, brains of P10 PLP-EGFP pups were dissected out into cold Gey's balanced salt solution containing 5 mg/ml glucose (GBSS-Glu) and meninges were removed. Cerebellar parasagittal slices (350 μm thick) were cut on a MacIlwain tissue chopper and transferred onto membranes of 30 mm Millipore culture inserts with 0.4 μm pore size. Slices were maintained in culture in six-well plates containing 1 ml of culture medium at 35°C in a 5% CO_2_ atmosphere. The medium was composed of 50% basal medium with Earle's salts, 25% Hanks' balanced salts solution, 25% horse serum, L-glutamine (1 mM) and 5 mg/ml glucose. The medium was changed every 2 days. Fourteen days later, cultures were fixed for one hour with 4% PFA for later immunostaining.

### Immunofluorescence analysis

Free-floating sections and cultured slices were processed identically for immunostaining. Slices were blocked with 0.1 M lysine solution in a PBS-GT buffer (PBS buffer supplemented with gelatin and 0.25% Triton 100X) for one hour and then incubated over night at 4°C with primary antibodies. Slices were then washed before being incubated with the corresponding secondary antibodies for two hours at room temperature. Processed slices were permanently mounted and pictures were taken using a Zeiss confocal microscope. For each cerebellar slice culture, three different cerebellar lobules were photographed. In brain sections, splenium, center and genu of corpus callosum were photographed. For each animal, images were analyzed using ImageJ software (NIH) to count oligodendrocyte cell numbers or to quantify the staining density (area %) in the case of MBP staining. For statistical analysis, we used the mean value of the three images per animal.

The following primary antibodies were used: anti-Olig2 (rabbit polyclonal; mouse monoclonal), anti-MBP (rabbit polyclonal; mouse monoclonal; rat monoclonal), anti-Adenomatous Polyposis Coli (CC1) (mouse monoclonal), anti-Calbindin D-28K (rabbit polyclonal), anti-Caspr (rabbit polyclonal), anti-Neurofilament 200 (NF200) (rabbit polyclonal). The following secondary antibodies were used: anti-mouse Alexa488 conjugated, anti-mouse Alexa633 conjugated anti-rabbit Alexa633 conjugated, anti-rabbit Cy3 conjugated, anti-rat Cy3 conjugated. A list of antibodies used can be found in the antibodies section of the key resources and identifiers (see below).

### Electron microscopy

Mice were perfused with phosphate buffer containing 2% PFA and 2% glutaraldehyde (Fluka). Tissues were dissected and immersed in the same fixative solution at 4°C overnight, washed in phosphate buffer, postfixed in 2% osmium tetroxide, dehydrated in graded ethanol series, and embedded in epoxy resin. Semithin sections of corpus callosum (genu) were cut with a glass knife at (0.5–1 μm) and stained with methylene blue/azur II.

Blocks were cut with 60-nm-thickness, and were stained with 3% uranyl acetate and 0.5% lead citrate. Ultrastructural analyses were performed in a JEOL jem-1011 electron microscope, equipped with a Gatan digital camera. Image acquisition was performed at the Cochin Imaging Facility. The g ratio, the ratio between the axon diameter and fiber diameter (axon diameter + myelin sheath) was measured using ImageJ software. To evaluate axon number, myelination density and g-ratio, at least 100 axonal cross sections were evaluated for each animal at higher magnification, x15000 micrographs (n ≥ 3 animals per group).

### Quantitative RT-PCR

At least four animals per group were sacrificed by decapitation. Brains were dissected out and the two hemispheres were separated and snap frozen using dry ice for further processing.

For qPCR analysis, RNA extraction from the left hemisphere was done using RNeasy Mini kit (Cat. No.217004, Qiagen, France) according the manufactures' instructions on the left hemisphere. For mRNA quantitation, Reverse Transcription (RT) was performed using High Capacity cDNA Reverse Transcription Kit (Part No 4368814, Applied Biosystems, UK), according to manufacturer’s instructions. Quantitative real-time PCR (qPCR) was performed using Power SYBR-Green Master Mix (ref 4367659, Applied Biosystems, UK) on an Applied 7300 Real-Time PCR system.

Primers used were:

MBP (forward, 5'-GTACAAGGACTCACACACGAGAACTAC-3';

reverse, 5’-TTGAAGAAATGGACTACTGGGTTTT -3’),

AR (forward, 5'-GACATGCGTTTGGACAGTACCA-3';

reverse, 5’-TCCACAGATCAGGCAGGTCTT-3’),

GAPDH (forward, 5'-GTCGGTGTGAACGGATTTGG-3';

reverse, 5’-GACTCCACGACATACTCAGC-3’),

CyclophilinA (cycloA) (forward, 5'-GTCAACCCCACCGTGTTCTT-3';

reverse, 5’-CTGCTGTCTTTGGGACCTTGT-3’).

### Western blotting

Tissue from the right hemisphere was homogenized in cold RIPA buffer plus protease inhibitor cocktail (Roche). Total protein concentration was measured using the BCA method (Pierce, Thermoscientific). Proteins (10 μg) were separated using a 12% polyacrylamide gel, followed by blotting onto a PVDF transfer membrane. Blots were incubated with rabbit antibody anti-myelin basic protein (MBP, 1:2000, AB980, Millipore) and with mouse antibody anti-β-actin (A5441, 1:5000, Sigma) and HRP-conjugated secondary antibodies (A2304 and A0545, 1:10000, Sigma). Densitometry quantification was done using ImageJ software (NIH).

### Measurement of steroid levels by GC-MS/MS

Animals were sacrificed by decapitation. Individual brains (60–100 mg) from P0, P5 and P10 male and female mice (at least n = 7 mice) were dissected out and the cerebella separated, weighted and stored at -20°C for further processing.

Steroid levels were determined by GC-MS/MS. Extraction was performed by adding 10 volumes of methanol and internal standards were introduced for steroid quantification. Samples were purified and fractionated by solid-phase extraction with the recycling procedure. The unconjugated steroid-containing fraction was filtered and further purified and fractionated by HPLC (Thermoscientific, USA). All the fractions were derivatized and analyzed by GC-MS/MS with an AI 1310 autosampler (Thermo Fisher Scientific, USA). The Trace 1310 gas chromatograph is coupled with a TSQ 8000 mass spectrometer (Thermo Fisher Scientific,USA). The mass spectrometer was used in tandem mode using Argon as collision gas. Injection was performed in the splitless mode at 250°C and the temperature of the gas chromatograph oven was initially maintained at 50°C for 1 min and ramped between 50 to 200°C at 20°C/min, then ramped to 300°C at 10°C/min and finally ramped to 350°C at 30°C/min. The helium carrier gas flow was maintained constant at 1 ml/min during the analysis. The transfer line and ionization chamber temperatures were 330°C and 200°C, respectively.

Electron impact ionization was used for mass spectrometry with ionization energy of 70 eV. Identification of steroids was supported by their retention time and according to two or three transitions. Quantification was performed according to the more abundant transition for the calibration solutions and for the biological extracts. The analytical protocol has been validated for all steroids by using a pool of male rat brain. The precision was in the 95–105% range, the limits of quantification of 0.002 ng/g and the interassay variation of 5–10%.

### Key resources and identifiers

#### Antibodies

from Millipore: Rabbit anti-Olig2, Cat# AB9610; RRID: AB_10141047; Mouse anti-Olig2, Cat# MABN50; RRID: AB_10807410; Rabbit anti-MBP, Cat# AB980; RRID: AB_92396; Mouse anti-MBP, Cat# MAB381; RRID: AB_94970; Rat anti-MBP, Cat# MAB386; Clone 12; RRID: AB_94975; Mouse anti- Adenomatous Polyposis Coli (CC1), Cat# OP80; RRID: AB_213434; Rabbit anti-Calbindin D-28K, Cat# CB-38; RRID: AB_10000340; Rabbit anti-Caspr, Cat# ab34151; RRID: AB_869934; Rabbit anti-Neurofilament 200 (NF200), Cat# MAB5256; RRID: AB_11210992; Anti-mouse Alexa488 conjugated, Cat# A11070; RRID: AB_142134; Anti-mouse Alexa633 conjugated, Cat# A-21053; RRID: AB_2535720; Anti-rat Cy3 conjugated, Cat# 111-165-144; RRID: AB_2338006; Mouse Anti-beta-Actin, Cat# A5441; clone AC-15; RRID: AB_476744.

#### Chemicals, peptides, and recombinant proteins

Paraformaldehyde (VWR, Cat# 28794.295); Glutaraldehyde (Fluka, Cat# G5882); Osmium Tetroxide (COGER, Cat# 31253.02); Cacodylate sodium (Fisher Scientific, Cat# BP325-50); BME (Dominique Dutscher, Cat# L0042-500); RnaseOut (Fisher, Cat# 10154652); DreamTaq DNA polymerase (Fisher, Cat# 15302445); L-Glutamine (GIBCO, Cat# 25030–024); Horse serum (GIBCO, Cat# 26050–088); HBSS (GIBCO, Cat# 14175–053); GEY’S Balanced Salt Solution (Sigma, Cat# G9779); Fluoromount (Clinisciences, Cat# 0100–01); Testosterone (Sigma, Cat# 86500); 5α-Dihydrotestosterone (5α-DHT) (Sigma, Cat# A8380-1G); Flutamide (Fluka, Cat# F9397-5G); Sesame oil (Sigma, Cat# S35547).

#### Critical commercial assays

Taqman gene expression master mix (Applied Biosystem, Cat# 4369514); High capacity cDNA RT KIT (Applied Biosystem, Cat# 10400745); RNeasy Mini kit (Qiagen, Cat# 217004); Power SYBR-Green Master Mix (Applied Biosystems, Cat# 4367659).

#### Experimental models

C57BL/6J mice (The Jackson Laboratory, Cat# JAX: 000664); PLP-EGFP mice (University of Colorado; Anschutz Medical Campus, Aurora, CO 80045. (Pr. Wendy Macklin) [23]; strain: B6/CBA (a cross between C57BL/6J females (B6) and CBA/J males (CBA) from The Jackson Laboratory); ARlox mice (genetic mixed background of C57BL/6 and 129SvEv, IGBMC, Strasbourg, France) [70]; Nestin cre mice (Genetic mixed background of C57BL/6 and 129SvEv, UPMC, France) [34]; ARNesCre mice (Local production); Tfm mice (French Atomic Energy Commission [41], Strain: (C57BL/6J-Aw-J.Cg-EdaTa-6J+/+ArTfm, Tfm/Y) (The Jackson Laboratory, Bar Harbor, ME); Millicel (MERCK, Cat# PICM03050);

#### Software and algorithms

ImageJ1.49g (NIH, USA, http://imagej.nih.gov/ij; java 1.6.0_14 (32-bit) 3011k of 1599MB; RRID: SCR_003070); Prism 6 (GraphPad, https://www.graphpad.com/scientific-software/prism/; RRID: SCR_002798); LSM software (Zeiss Microscope, https://www.zeiss.com/microscopy/int/products/microscope-software/zen.html; RRID: SCR_014344); Adobe Illustrator CC (Adobe, http://www.adobe.com/products/illustrator.html; RRID: SCR_010279).

#### Others

Macllwain tissue chopper (WPI, Cat# 95060–164); Vibratome (Leica, Cat# VT1000S); Zeiss confocal microscope (Cat# LSM510-Meta); Transmission electron Microscope (1011 JEOL (JEOL, Cat# 1011 JEOL); Applied 7300 Real-Time PCR system (Applied Biosystems, Cat# 4351103); HPLC (Thermoscientific, USA, Cat# Ultimate3000 RSLCnano System); GC-MS/MS with an AI 1310 Autosampler The Trace1310 (Thermo Fisher Scientific, USA, Cat# AI 1310; The Trace 1310); Gas chromatograph is coupled with a TSQ 8000 mass spectrometer (Thermo Fisher Scientific, USA, Cat# TSQ8000).

#### Statistical analysis

Statistical parameters including the definitions and exact value of n, deviations, p values, and the types of the statistical tests are reported in the corresponding figure legends. Statistical analysis was carried out using Prism 6 (GraphPad Software). All statistical comparisons were conducted on data originating from at least three or more biologically independent experimental samples. Statistical comparisons between two groups were performed by an unpaired t test. Comparisons between three or more groups were performed by two-way ANOVA or one-way ANOVA followed by Newman-Keuls tests for pairwise comparisons. Data are expressed as means ± SEM. Differences were considered significant with p < 0.05.
